# Association between *TERT* gene polymorphisms and acute myeloid leukemia susceptibility in a Chinese population: a case–control study

**DOI:** 10.1186/s12935-020-01335-3

**Published:** 2020-07-16

**Authors:** Yong Tong, Yinzhou Xiang, Bao Li, Shijie Bao, Ying Zhou, Wen Yuan, Yu Ling, Dan Hao, Huamin Zhu, Zhiqiang Sun

**Affiliations:** 1 Department of Hematology, Shenzhen Hospital, Southern Medical University, Shenzhen, 518000 Guangdong China; 2 Department of Otolaryngology, China Three Gorges University People’s Hospital, Yichang, 443000 Hubei China; 3 Department of Hematopathology, The University of Hong Kong-Shenzhen Hospital, Shenzhen, 518000 Guangdong China

**Keywords:** *TERT*, AML, Susceptibility, PCR-RFLP

## Abstract

**Background:**

The aim of this study was to investigate the association between telomerase reverse transcriptase (*TERT*) gene polymorphisms and acute myeloid leukemia (AML) susceptibility in a Chinese Han population.

**Methods:**

A total of 102 AML patients and 108 healthy controls were enrolled in this case–control study. *TERT* gene rs2853669 and rs2736100 polymorphisms were genotyped via polymerase chain reaction-restriction fragment length polymorphism (PCR-RFLP). Chi-square test was applied to compare polymorphism distributions between case and control groups. The strength of the association between *TERT* gene polymorphisms and AML susceptibility was evaluated utilizing odds ratio (OR) with corresponding 95% confidence interval (CI).

**Results:**

CC genotype and C allele of rs2736100 polymorphism were more frequent in AML patients (*P *< 0.05), and individuals carrying CC genotype showed higher risk of suffering from AML (OR = 2.632, 95% CI 1.129–6.133). But for rs2853669 polymorphism, no significant differences were detected in either genotype or allele distributions between groups (*P *> 0.05).

**Conclusions:**

This study suggested a positive association between *TERT* gene rs2736100 polymorphism and AML susceptibility in Chinese Han population.

## Background

Acute myeloid leukemia (AML) is a malignant disorder of clonal hematopoietic stem cell, and has been considered as the most common acute leukemia affecting adults [1]. AML is characterized by rapid proliferation of leukemic blasts [2]. It can result in abnormal accumulation of immature cells and the suppression of normal hematopoiesis [3, 4]. Several environmental factors have been identified to be involved in AML development, such as benzene exposure, ionizing radiation, and chemotherapy [5–7]. But not all individuals exposing to the same environmental risk factors will develop AML. Hence, genetic factors are generally accepted to play crucial roles in the pathogenesis of AML.

Telomerase reverse transcriptase (abbreviated to TERT, or hTERT in humans) is a catalytic subunit of telomerase, and together with telomerase RNA component (TERC), constitutes the most important unit of telomerase complex [8]. TERT plays an important role in the maintenance of telomeres, chromosome stability and preventing malignancy [9]. Abnormal activity of telomerase has been reported to be involved in cancer initiation and development, and telomere length shows negative association with cancer incidence and mortality [10].

Human *TERT* gene is located on chromosome 5p15.33, and several single nucleotide polymorphisms (SNPs) have been identified in this gene. Up to now, numbers of studies have discussed association between *TERT* gene SNPs and the risks of various cancers, including breast, lung, colorectal, ovarian, prostate, and gastric cancers [11, 12]. Recently, Mosrati et al. [13] have explored the association between *TERT* polymorphisms and AML susceptibility, focusing on several SNPs, among which only two SNPs (rs2853669 and rs2736100) showed significant association. But genetic association has not been reported in Chinese Han population.

Therefore, a replication was conducted in this paper, and the association of *TERT* gene rs2853669 and rs2736100 polymorphisms with AML susceptibility was investigated in Chinese Han population.

## Methods

### Subjects

This case–control study was approved and consented by Ethics committee of Fujian Provincial Hospital, Provincial Clinical Medical College of Fujian Medical University, and protocol number from the Ethic committee was 2019(45). Sample collection was in compliance with the ethnic criteria of national human genome research. Participants involved in this paper all agreed to provide blood samples and receive investigation, and they or their guardians signed informed consent before enrollment. All subjects were Chinese Han population and had no blood relationship with each other.

A total of 210 individuals were enrolled in this study, including 102 AML patients and 108 healthy controls. All cases were first diagnosed with AML at Fujian Provincial Hospital, Provincial Clinical Medical College of Fujian Medical University. AML cases were diagnosed according to World Health Organization criteria, namely increased number of myeloblasts in bone marrow or peripheral blood, and the disease would be confirmed when a 200-cell differential revealed the presence of 20% or more myeloblasts in a marrow aspirate or in blood [14]. With the following conditions, AML cases would be excluded from the current study: cancer history, blood disorders, diabetes, and connective tissue disease. Another 108 healthy participants were recruited into control group, who visited the same hospital for routine health check-ups during the same period. The individuals in control group were healthy, and had no evidences for family history of blood diseases, cancers, chronic diseases, connective tissue disease, etc. Control group was matched with the case group in age, gender, body mass index (BMI) and life conditions.

### DNA extraction

Blood sample of each participant was collected at diagnosis before treatment initiation. Genomic DNA was isolated from blood samples using TaKaRa Genome DNA Extraction Kit (Dalian Biological Engineering CO., LTD, China) according to manufacturer’s instructions. Extracted DNAs were solved in sterile distilled water and stored at − 20 °C for standby application.

### Genotyping

Polymerase chain reaction-restriction fragment length polymorphism (PCR-RFLP) method was applied for the genotyping of *TERT* gene rs2853669 and rs2736100 polymorphisms. Primer sequences for the SNPs were designed by Primer Premier 5.0, and synthesized by Sangon Biotech (Shanghai, China) (Table 1). PCR procedures consisted of an initial degeneration at 95 °C for 5 min, followed by 30 cycles of 94 °C for 30 s, annealing at 55 °C for 30 s, and extension at 72 °C for 30 s, and a final extension at 72 °C for 7 min. Reaction without DNA sample was employed as negative control.

**Table 1:** Primer sequences of *TERT* gene rs2853669 and rs2736100 polymorphisms

SNP	Primer sequences	
rs2853669	Sense	5′-CAGCGCTGCCTGAAACTC-3′
	Reverse	5′-GTCCTGCCCCTTCACCTT-3′
rs2736100	Sense	5′-CCCCACAAGCTAAGCATTAT-3′
	Reverse	5′-GAAGAACCACGCAAAGGAC-3′

PCR production was detected through 1.5% agarose gel electrophoresis. Then each successful PCR product was digested by 10U restriction endonuclease enzymes (Shanghai Keith Tell biological science and Technology Co., Ltd.). Two enzymes *SacI* and SfcI were applied for the digestion of PCR productions of rs2853669 and rs2736100 polymorphisms, respectively. Digestion reactions were performed at 37 °C overnight, and digested fragments were electrophoresed on 3% agarose gel containing 0.5 μg/mL ethidium bromide, and visualized under UV illumination. Digestion reaction without PCR production was adopted as negative control. In addition, 20% of amplification results were randomly selected and sent for direct sequencing to estimate the accuracy of digestion results.

### Statistical analysis

PASW statistics 18.0 statistical software was applied for data analysis in this paper. Genotype and allele frequencies of *TERT* gene rs2853669 and rs2736100 polymorphisms were estimated via direct counting and expressed in percentage (%). Hardy–Weinberg equilibrium (HWE) for each polymorphism in control group was analyzed to assess the quality of the study subjects. Distribution differences of each polymorphism were compared between groups via Chi-square test. Strength of association between *TERT* gene polymorphisms and AML susceptibility was evaluated adopting odds ratios (OR) with 95% confidence interval (CI). Two sided *P*-values of less than 0.05 were considered as statistically significant level.

## Results

### Baseline characteristics of the study subjects

There were 102 AML cases including 48 males and 54 females who were aged 33–64, with an average age of 41.23 ± 3.45 years. The control group contained 108 healthy individuals, including 52 and 56 females, and they were aged 31–68 years, with an average age of 40.43 ± 4.07 years. The control and case groups were matched in age and gender (*P *> 0.05 for both). BMI value was 23.32 ± 2.29 kg/m^2^ in case group and 22.19 ± 1.96 kg/m^2^ in control group was, without significant difference (*P *= 0.712). The distributions of blood white blood count (WBC) were significantly different between case and control groups (*P *< 0.001). Baseline characteristics of case and control groups were summarized in Table 2.

**Table 2:** The detailed information of subjects in cases

Characteristics	AML group	Healthy controls	*P* values
Mean age (years)	41.27 ± 3.45	40.43 ± 4.07	0.367
Gender			0.874
Male	48 (47.06)	52 (48.15)	
Female	54 (52.94)	56 (51.85)	
BMI (kg/m^2^)	23.32 ± 2.29	22.19 ± 1.96	0.712
WBC (count × 10^9^/L)			< 0.001
< 4	39 (38.24)	5 (4.63)	
4–10	13 (12.74)	79 (73.15)	
> 10	50 (49.02)	16 (14.81)	
FAB classification		–	–
M0	2 (1.96)		–
M1	4 (3.92)		
M2	21 (20.59)		
M3	32 (31.37)		
M4	19 (18.63)		
M5	15 (14.71)		
M6	5 (4.90)		
M7	4 (3.92)		
Immunophenotype		–	–
T cell type	21 (20.59)		
Pre-B cell type	81 (79.41)		
Lymphadenopathy			–
Yes	20 (19.61)	0 (0.00)	
No	82 (80.39)	108 (100.00)	

### HWE test

Genotype and allele frequencies of the interested polymorphisms were analyzed through PCR-RFLP method, and the results were in line with sequencing results. All genotypes of *TERT* gene rs2853669 and rs2736100 SNPs conformed to HWE Law in controls (Table 3, *P *> 0.05), suggesting the representativeness of the study sample.

**Table 3:** Genotype and allele distributions of *TERT* gene rs2853669 and rs2736100 polymorphisms in case and control groups

Genotype/allele	Case *n *= 102 (%)	Control *n *= 108 (%)	$\chi^{2}$	P	OR (95% CI)
rs2853669					
AA	25 (24.51)	36 (33.33)	–	–	1
AG	56 (54.90)	59 (54.63)	0.954	0.329	1.367 (0.730–2.561)
GG	21 (20.59)	13 (12.04)	3.775	0.052	2.326 (0.985–5.494)
A	106 (51.96)	131 (60.65)	–	–	1
G	98 (48.04)	85 (39.35)	3.220	0.073	1.425 (0.967–2.099)
*P*^*HWE*^		0.133			
rs2736100					
AA	19 (18.63)	32 (29.63)	–	–	1
AC	58 (56.86)	60 (55.56)	2.032	0.154	1.628 (0.831–3.190)
CC	25 (24.51)	16 (14.81)	5.125	0.024	2.632 (1.129–6.133)
A	96 (47.06)	124 (57.41)	–	–	1
C	108 (52.94)	92 (42.59)	4.504	0.034	1.516 (1.032–2.229)
*P*^*HWE*^		0.157			

### Association of *TERT* SNPs with AML

For rs2853669 polymorphism, GG genotype increased remarkably in case group (20.59% vs 12.04%), while AA genotype frequency decreased in cases (24.51% vs 33.33%), but the differences did not reach significant level (*P *> 0.05). G allele frequency also showed a decreasing trend in the case group (48.04% vs 39.35%), but the difference was not statistically significant (*P *> 0.05). The results suggested that *TERT* gene rs2853669 polymorphism might have no obvious association with AML susceptibility (Table 3).

Significant differences were detected for rs2736100 polymorphism in both genotype and allele distributions between case and control groups. Specifically, CC genotype was more frequent in case group than in controls (24.51% vs 14.81%), while AA genotype frequency was less in case group (18.63% vs 29.63%), and the differences reached significant level (*P *= 0.024). Compared with AA genotype carriers, individuals carrying CC genotype showed 2.632 folds higher risk to suffer from AML (OR = 2.632, 95% CI 1.129–6.133). AC genotype showed no obvious distribution differences between case and control groups (56.86% vs 55.56%, *P *> 0.05). Besides, rs2736100 A and C allele frequencies were 47.06%, 52.94% in case group and 57.41%, 42.59% in control group, with C allele significantly increasing in case group (*P *= 0.034). Rs2736100 C allele carriers showed higher risk to suffer from AML (OR = 1.516, 95% CI 1.032–2.229). All results suggested that *TERT* gene rs2736100 polymorphism was positively associated with AML susceptibility in Chinese Han population, and CC genotype and C allele were risk factors for AML.

## Discussion

AML is a fatal hematopoietic stem cell tumor with poor outcome, inhibiting normal hematopoiesis [15]. For AML patients, early detection and timely treatment can increase survival chances, and cytogenetic and molecular analyses play crucial roles in predicting remission and survival rates [16]. But it is difficult to estimate AML prognosis due to diverse molecular mechanisms of the disease. Therefore, it is necessary to find more effective biomarkers.

Unlimited proliferation is an important characteristic of cancer cells, and the activation of telomerase is a key process to achieve this characteristic in the vast majority of various cancers, including AML [17, 18]. *TERT* gene encodes a key catalytic subunit of telomerase, and maintains telomere stability. Gene expression disorder always brings about abnormal telomerase activation and further results in unlimited cell proliferation and even malignancies [19]. Considering central role of TERT in oncogenesis, numerous studies have discussed the association between cancer susceptibility and SNPs in *TERT*, and accumulated evidences have suggested significant association between two sides [20, 21].

In the present study, two SNPs (rs2853669 and rs2736100) in *TERT* gene were analyzed in 102 AML patients and 108 healthy controls, and significant association was detected between rs2736100 polymorphism and AML susceptibility in Chinese Han population. Significantly higher frequency of rs2736100 CC genotype was detected in AML patients, and individuals carrying CC genotype showed 2.632 folds higher risk of AML. We also found higher frequency of rs2736100 C allele in AML case group, suggesting C allele assumed a promoting effect on the onset of AML. Rs2736100 polymorphism is located in the second intron of *TERT* gene. Its association with cancer susceptibility has been investigated extensively. Recently, a study has suggested that rs2736100 polymorphism regulated TERT expression and telomere length in gastric cancer patients [22]. As reported by previous study, rs2736100 CC genotype increased the risk of various cancers, such as bladder, lung and pancreas cancer, but decreased testicular cancer risk [23]. Furthermore, Mosrati et al. [13] have suggested that *TERT* gene rs2736100 polymorphism significantly increased AML risk in Swedish population. In the present study, *TERT* gene rs2736100 polymorphism showed positive association with the susceptibility to AML in Chinese Han population, which was in accordance with previous evidences. Besides, rs2736100 CC genotype has been reported to be significantly associated with the upregulation of TERT expression through which its oncogenic effect was exerted [24]. But exact mechanism of rs2736100 polymorphism affecting AML susceptibility has not been explored. Rs2853669 polymorphism is located at − 245 bp from *TERT* ATG site. Several cancers have been associated with rs2853669 polymorphism, such as breast cancer and lung cancer [25, 26], though relevant results remain inconclusive. Significant association has also been detected between rs2853669 polymorphism and AML susceptibility in a Swedish population [13], but no significant association was detected in Chinese Han population. The difference might be caused by different genetic backgrounds.

Several limitations still presented in this paper which needed to be addressed. Firstly, our study conformed to candidate gene approach, and only one gene and two polymorphisms were involved in the study. Secondly, the sample size was relatively small, which may influence statistical power. Thirdly, gene-environment interaction was not evaluated. Besides, the causality between *TERT* SNPs and AML susceptibility was not certified in this study. In addition, genetic interactions between different SNPs in *TERT* gene in AML development were not explored. Thus, more studies with enlarged sample sizes are required to check the impact of *TERT* gene polymorphisms on AML susceptibility.

## Conclusions

In conclusion, positive association was discovered between *TERT* gene rs2736100 polymorphism and AML susceptibility in Chinese Han population, and CC genotype was confirmed as a risk factor for AML. Although significant association was detected, more researches are needed to verify the results.

## Data Availability

The datasets used and/or analyzed during the current study are available from the corresponding author on reasonable request.

## Abbreviations

*TERT*: Telomerase reverse transcriptase
AML: Acute myeloid leukemia
PCR-RFLP: Polymerase chain reaction-restriction fragment length polymorphism
OR: Odds ratio
CI: Confidence interval
AML: Acute myeloid leukemia
TERC: Telomerase RNA component
SNPs: Single nucleotide polymorphisms
BMI: Body mass index
